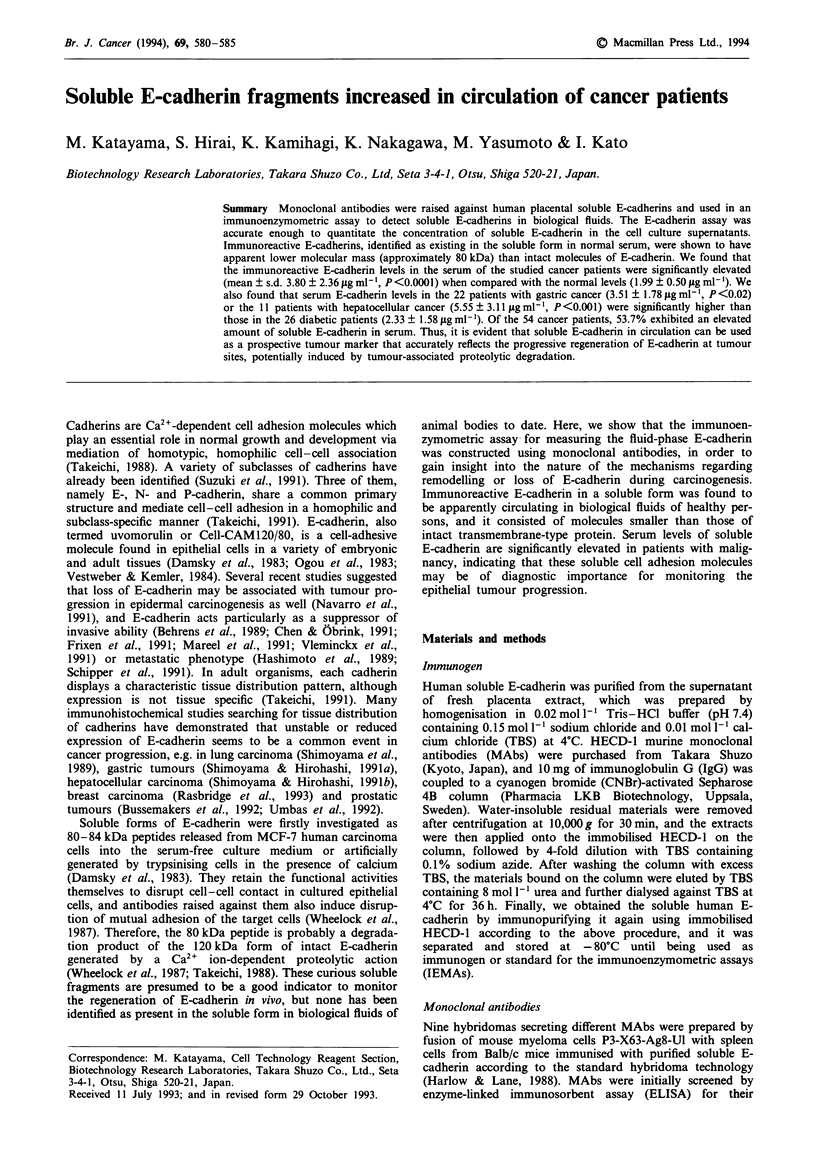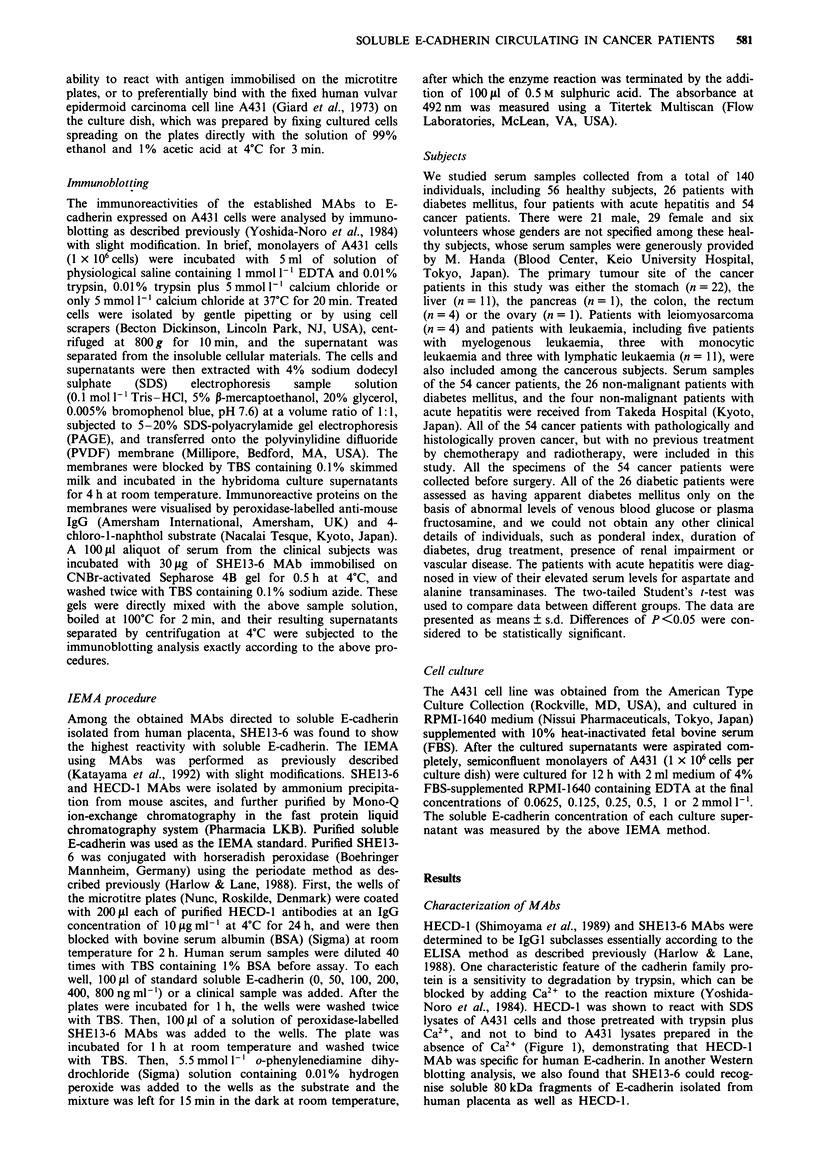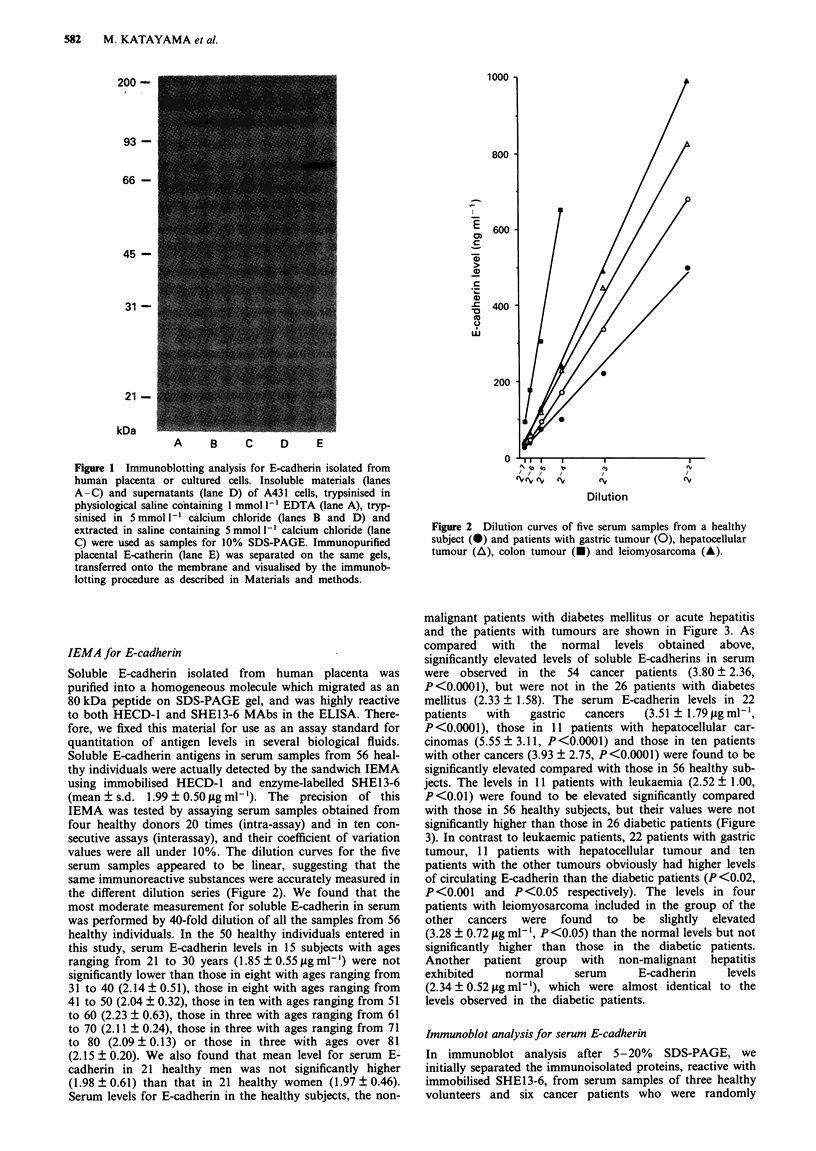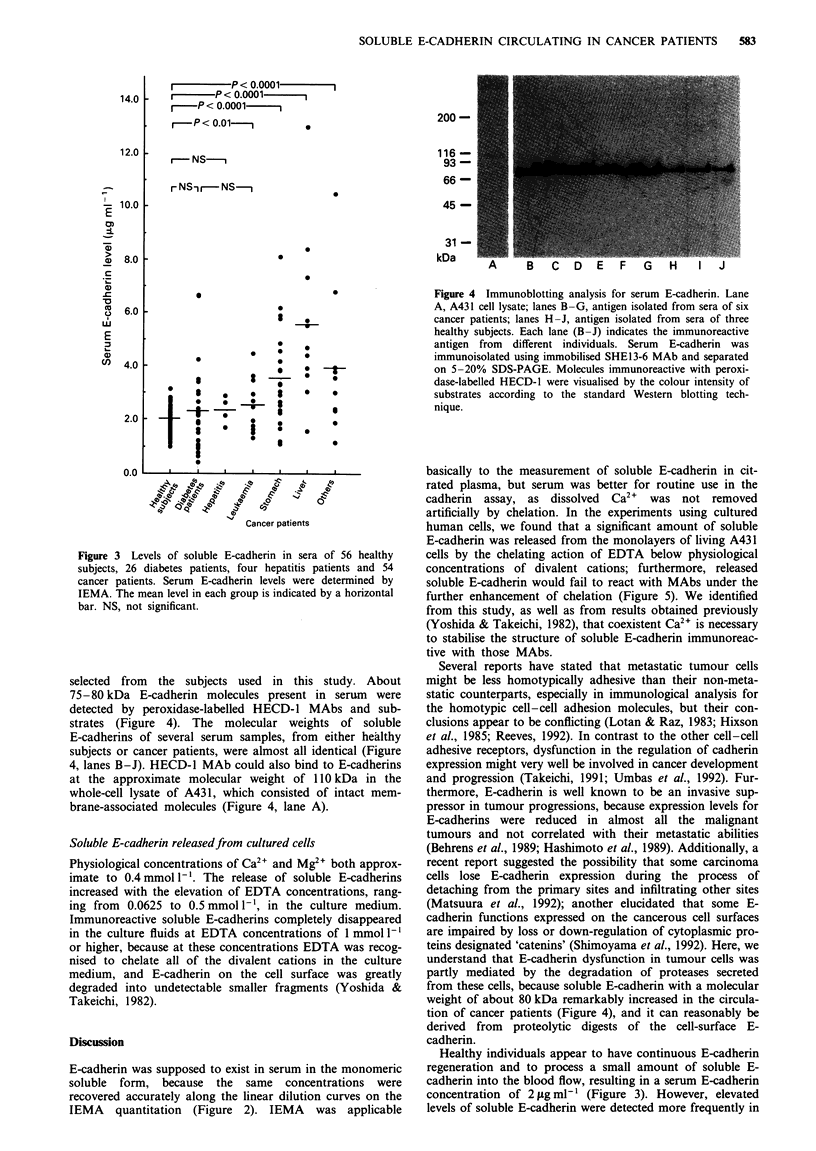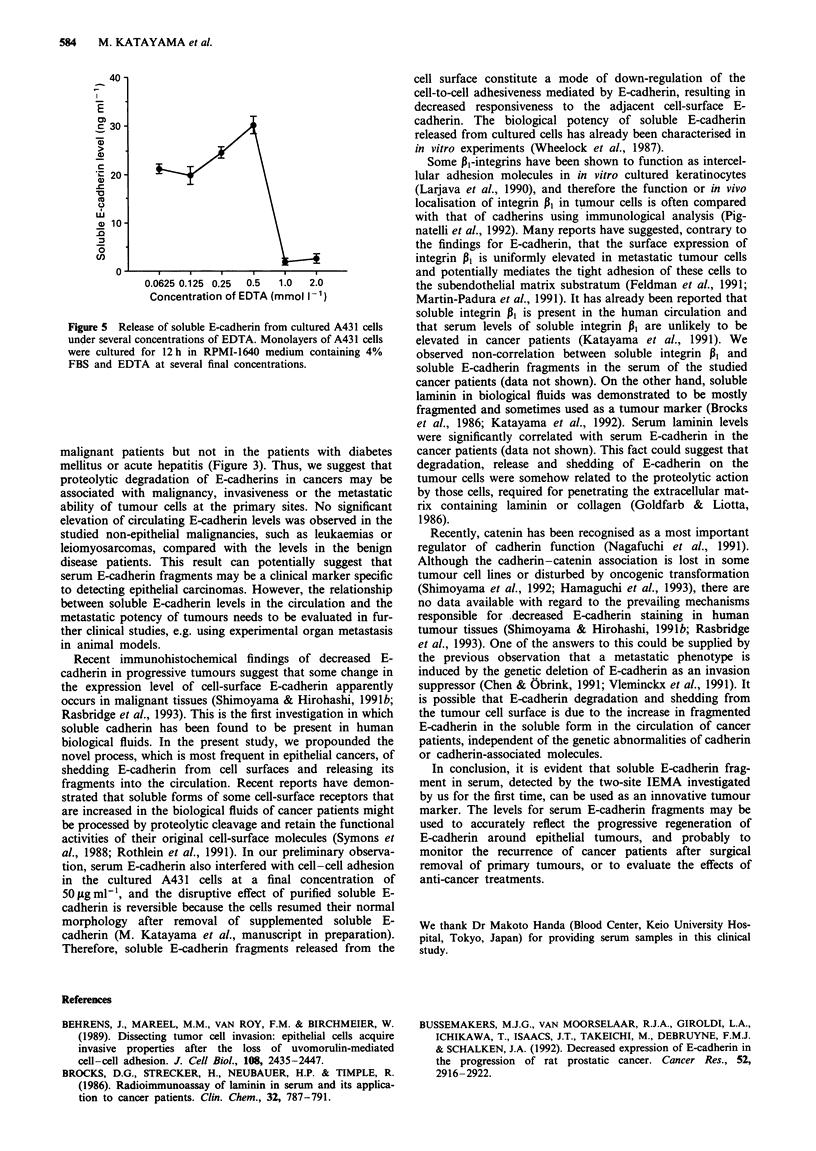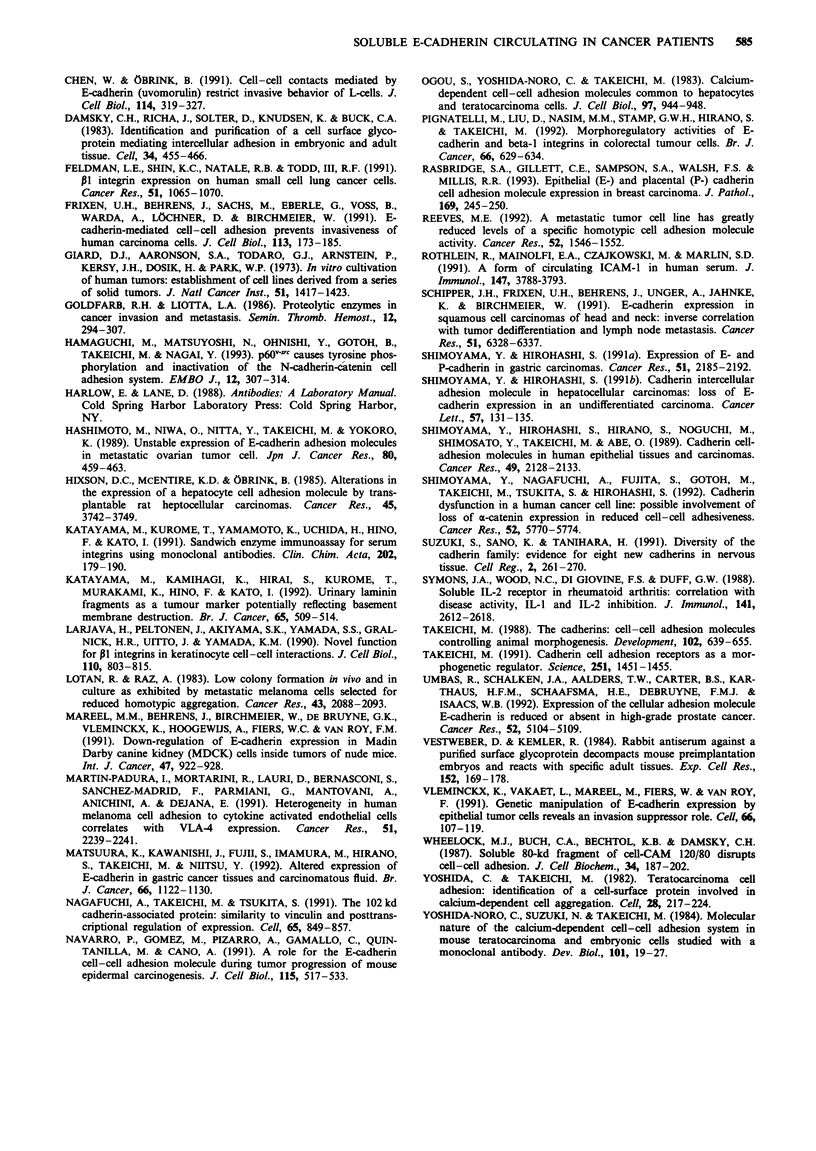# Soluble E-cadherin fragments increased in circulation of cancer patients.

**DOI:** 10.1038/bjc.1994.106

**Published:** 1994-03

**Authors:** M. Katayama, S. Hirai, K. Kamihagi, K. Nakagawa, M. Yasumoto, I. Kato

**Affiliations:** Biotechnology Research Laboratories, Takara Shuzo Co., Ltd., Shiga, Japan.

## Abstract

**Images:**


Br. J. Cancer (1994), 69, 580-585 © Macmillan Press Ltd., 1994

### Soluble E-cadherin fragments increased in circulation of cancer patients

M. Katayama, S. Hirai, K. Kamihagi, K. Nakagawa, M. Yasumoto & I. Kato

*Biotechnology Research Laboratories, Takara Shuzo Co., Ltd, Seta 3-4-1, Otsu, Shiga 520-21, Japan.*

**Summary** Monoclonal antibodies were raised against human placental soluble E-cadherins and used in an immunoenzymometric assay to detect soluble E-cadherins in biological fluids. The E-cadherin assay was accurate enough to quantitate the concentration of soluble E-cadherin in the cell culture supernatants. Immunoreactive E-cadherins, identified as existing in the soluble form in normal serum, were shown to have apparent lower molecular mass (approximately 80 kDa) than intact molecules of E-cadherin. We found that the immunoreactive E-cadherin levels in the serum of the studied cancer patients were significantly elevated (mean ± s.d. 3.80 ± 2.36 $\mu g\ ml^{-1}$, $P<0.0001$) when compared with the normal levels (1.99 ± 0.50 $\mu g\ ml^{-1}$). We also found that serum E-cadherin levels in the 22 patients with gastric cancer (3.51 ± 1.78 $\mu g\ ml^{-1}$, $P<0.02$) or the 11 patients with hepatocellular cancer (5.55 ± 3.11 $\mu g\ ml^{-1}$, $P<0.001$) were significantly higher than those in the 26 diabetic patients (2.33 ± 1.58 $\mu g\ ml^{-1}$). Of the 54 cancer patients, 53.7% exhibited an elevated amount of soluble E-cadherin in serum. Thus, it is evident that soluble E-cadherin in circulation can be used as a prospective tumour marker that accurately reflects the progressive regeneration of E-cadherin at tumour sites, potentially induced by tumour-associated proteolytic degradation.

Cadherins are $Ca^{2+}$-dependent cell adhesion molecules which play an essential role in normal growth and development via mediation of homotypic, homophilic cell–cell association (Takeichi, 1988). A variety of subclasses of cadherins have already been identified (Suzuki *et al.*, 1991). Three of them, namely E-, N- and P-cadherin, share a common primary structure and mediate cell–cell adhesion in a homophilic and subclass-specific manner (Takeichi, 1991). E-cadherin, also termed uvomorulin or Cell-CAM120/80, is a cell-adhesive molecule found in epithelial cells in a variety of embryonic and adult tissues (Damsky *et al.*, 1983; Ogou *et al.*, 1983; Vestweber & Kemler, 1984). Several recent studies suggested that loss of E-cadherin may be associated with tumour progression in epidermal carcinogenesis as well (Navarro *et al.*, 1991), and E-cadherin acts particularly as a suppressor of invasive ability (Behrens *et al.*, 1989; Chen & Öbrink, 1991; Frixen *et al.*, 1991; Mareel *et al.*, 1991; Vleminckx *et al.*, 1991) or metastatic phenotype (Hashimoto *et al.*, 1989; Schipper *et al.*, 1991). In adult organisms, each cadherin displays a characteristic tissue distribution pattern, although expression is not tissue specific (Takeichi, 1991). Many immunohistochemical studies searching for tissue distribution of cadherins have demonstrated that unstable or reduced expression of E-cadherin seems to be a common event in cancer progression, e.g. in lung carcinoma (Shimoyama *et al.*, 1989), gastric tumours (Shimoyama & Hirohashi, 1991*a*), hepatocellular carcinoma (Shimoyama & Hirohashi, 1991*b*), breast carcinoma (Rasbridge *et al.*, 1993) and prostatic tumours (Bussemakers *et al.*, 1992; Umbas *et al.*, 1992).

Soluble forms of E-cadherin were firstly investigated as 80–84 kDa peptides released from MCF-7 human carcinoma cells into the serum-free culture medium or artificially generated by trypsinising cells in the presence of calcium (Damsky *et al.*, 1983). They retain the functional activities themselves to disrupt cell–cell contact in cultured epithelial cells, and antibodies raised against them also induce disruption of mutual adhesion of the target cells (Wheelock *et al.*, 1987). Therefore, the 80 kDa peptide is probably a degradation product of the 120 kDa form of intact E-cadherin generated by a $Ca^{2+}$ ion-dependent proteolytic action (Wheelock *et al.*, 1987; Takeichi, 1988). These curious soluble fragments are presumed to be a good indicator to monitor the regeneration of E-cadherin *in vivo*, but none has been identified as present in the soluble form in biological fluids of animal bodies to date. Here, we show that the immunoenzymometric assay for measuring the fluid-phase E-cadherin was constructed using monoclonal antibodies, in order to gain insight into the nature of the mechanisms regarding remodelling or loss of E-cadherin during carcinogenesis. Immunoreactive E-cadherin in a soluble form was found to be apparently circulating in biological fluids of healthy persons, and it consisted of molecules smaller than those of intact transmembrane-type protein. Serum levels of soluble E-cadherin are significantly elevated in patients with malignancy, indicating that these soluble cell adhesion molecules may be of diagnostic importance for monitoring the epithelial tumour progression.

Correspondence: M. Katayama, Cell Technology Reagent Section, Biotechnology Research Laboratories, Takara Shuzo Co., Ltd., Seta 3-4-1, Otsu, Shiga 520-21, Japan.
Received 11 July 1993; and in revised form 29 October 1993.

### Materials and methods

### *Immunogen*

Human soluble E-cadherin was purified from the supernatant of fresh placenta extract, which was prepared by homogenisation in 0.02 $mol\ l^{-1}$ Tris-HCl buffer (pH 7.4) containing 0.15 $mol\ l^{-1}$ sodium chloride and 0.01 $mol\ l^{-1}$ calcium chloride (TBS) at 4°C. HECD-1 murine monoclonal antibodies (MAbs) were purchased from Takara Shuzo (Kyoto, Japan), and 10 mg of immunoglobulin G (IgG) was coupled to a cyanogen bromide (CNBr)-activated Sepharose 4B column (Pharmacia LKB Biotechnology, Uppsala, Sweden). Water-insoluble residual materials were removed after centrifugation at 10,000 *g* for 30 min, and the extracts were then applied onto the immobilised HECD-1 on the column, followed by 4-fold dilution with TBS containing 0.1% sodium azide. After washing the column with excess TBS, the materials bound on the column were eluted by TBS containing 8 $mol\ l^{-1}$ urea and further dialysed against TBS at 4°C for 36 h. Finally, we obtained the soluble human E-cadherin by immunopurifying it again using immobilised HECD-1 according to the above procedure, and it was separated and stored at −80°C until being used as immunogen or standard for the immunoenzymometric assays (IEMAs).

### *Monoclonal antibodies*

Nine hybridomas secreting different MAbs were prepared by fusion of mouse myeloma cells P3-X63-Ag8-U1 with spleen cells from Balb/c mice immunised with purified soluble E-cadherin according to the standard hybridoma technology (Harlow & Lane, 1988). MAbs were initially screened by enzyme-linked immunosorbent assay (ELISA) for their ability to react with antigen immobilised on the microtitre plates, or to preferentially bind with the fixed human vulvar epidermoid carcinoma cell line A431 (Giard *et al.*, 1973) on the culture dish, which was prepared by fixing cultured cells spreading on the plates directly with the solution of 99% ethanol and 1% acetic acid at 4°C for 3 min.

*Immunoblotting*

The immunoreactivities of the established MAbs to E-cadherin expressed on A431 cells were analysed by immunoblotting as described previously (Yoshida-Noro *et al.*, 1984) with slight modification. In brief, monolayers of A431 cells ($1 \times 10^6$ cells) were incubated with 5 ml of solution of physiological saline containing 1 mmol $l^{-1}$ EDTA and 0.01% trypsin, 0.01% trypsin plus 5 mmol $l^{-1}$ calcium chloride or only 5 mmol $l^{-1}$ calcium chloride at 37°C for 20 min. Treated cells were isolated by gentle pipetting or by using cell scrapers (Becton Dickinson, Lincoln Park, NJ, USA), centrifuged at 800 *g* for 10 min, and the supernatant was separated from the insoluble cellular materials. The cells and supernatants were then extracted with 4% sodium dodecyl sulphate (SDS) electrophoresis sample solution (0.1 mol $l^{-1}$ Tris-HCl, 5% $\beta$-mercaptoethanol, 20% glycerol, 0.005% bromophenol blue, pH 7.6) at a volume ratio of 1:1, subjected to 5-20% SDS-polyacrylamide gel electrophoresis (PAGE), and transferred onto the polyvinylidine difluoride (PVDF) membrane (Millipore, Bedford, MA, USA). The membranes were blocked by TBS containing 0.1% skimmed milk and incubated in the hybridoma culture supernatants for 4 h at room temperature. Immunoreactive proteins on the membranes were visualised by peroxidase-labelled anti-mouse IgG (Amersham International, Amersham, UK) and 4-chloro-1-naphthol substrate (Nacalai Tesque, Kyoto, Japan). A 100 µl aliquot of serum from the clinical subjects was incubated with 30 µg of SHE13-6 MAb immobilised on CNBr-activated Sepharose 4B gel for 0.5 h at 4°C, and washed twice with TBS containing 0.1% sodium azide. These gels were directly mixed with the above sample solution, boiled at 100°C for 2 min, and their resulting supernatants separated by centrifugation at 4°C were subjected to the immunoblotting analysis exactly according to the above procedures.

*IEMA procedure*

Among the obtained MAbs directed to soluble E-cadherin isolated from human placenta, SHE13-6 was found to show the highest reactivity with soluble E-cadherin. The IEMA using MAbs was performed as previously described (Katayama *et al.*, 1992) with slight modifications. SHE13-6 and HECD-1 MAbs were isolated by ammonium precipitation from mouse ascites, and further purified by Mono-Q ion-exchange chromatography in the fast protein liquid chromatography system (Pharmacia LKB). Purified soluble E-cadherin was used as the IEMA standard. Purified SHE13-6 was conjugated with horseradish peroxidase (Boehringer Mannheim, Germany) using the periodate method as described previously (Harlow & Lane, 1988). First, the wells of the microtitre plates (Nunc, Roskilde, Denmark) were coated with 200 µl each of purified HECD-1 antibodies at an IgG concentration of 10 µg $ml^{-1}$ at 4°C for 24 h, and were then blocked with bovine serum albumin (BSA) (Sigma) at room temperature for 2 h. Human serum samples were diluted 40 times with TBS containing 1% BSA before assay. To each well, 100 µl of standard soluble E-cadherin (0, 50, 100, 200, 400, 800 ng $ml^{-1}$) or a clinical sample was added. After the plates were incubated for 1 h, the wells were washed twice with TBS. Then, 100 µl of a solution of peroxidase-labelled SHE13-6 MAbs was added to the wells. The plate was incubated for 1 h at room temperature and washed twice with TBS. Then, 5.5 mmol $l^{-1}$ *o*-phenylenediamine dihydrochloride (Sigma) solution containing 0.01% hydrogen peroxide was added to the wells as the substrate and the mixture was left for 15 min in the dark at room temperature, after which the enzyme reaction was terminated by the addition of 100 µl of 0.5 M sulphuric acid. The absorbance at 492 nm was measured using a Titertek Multiscan (Flow Laboratories, McLean, VA, USA).

*Subjects*

We studied serum samples collected from a total of 140 individuals, including 56 healthy subjects, 26 patients with diabetes mellitus, four patients with acute hepatitis and 54 cancer patients. There were 21 male, 29 female and six volunteers whose genders are not specified among these healthy subjects, whose serum samples were generously provided by M. Handa (Blood Center, Keio University Hospital, Tokyo, Japan). The primary tumour site of the cancer patients in this study was either the stomach ($n = 22$), the liver ($n = 11$), the pancreas ($n = 1$), the colon, the rectum ($n = 4$) or the ovary ($n = 1$). Patients with leiomyosarcoma ($n = 4$) and patients with leukaemia, including five patients with myelogenous leukaemia, three with monocytic leukaemia and three with lymphatic leukaemia ($n = 11$), were also included among the cancerous subjects. Serum samples of the 54 cancer patients, the 26 non-malignant patients with diabetes mellitus, and the four non-malignant patients with acute hepatitis were received from Takeda Hospital (Kyoto, Japan). All of the 54 cancer patients with pathologically and histologically proven cancer, but with no previous treatment by chemotherapy and radiotherapy, were included in this study. All the specimens of the 54 cancer patients were collected before surgery. All of the 26 diabetic patients were assessed as having apparent diabetes mellitus only on the basis of abnormal levels of venous blood glucose or plasma fructosamine, and we could not obtain any other clinical details of individuals, such as ponderal index, duration of diabetes, drug treatment, presence of renal impairment or vascular disease. The patients with acute hepatitis were diagnosed in view of their elevated serum levels for aspartate and alanine transaminases. The two-tailed Student's *t*-test was used to compare data between different groups. The data are presented as means ± s.d. Differences of $P < 0.05$ were considered to be statistically significant.

*Cell culture*

The A431 cell line was obtained from the American Type Culture Collection (Rockville, MD, USA), and cultured in RPMI-1640 medium (Nissui Pharmaceuticals, Tokyo, Japan) supplemented with 10% heat-inactivated fetal bovine serum (FBS). After the cultured supernatants were aspirated completely, semiconfluent monolayers of A431 ($1 \times 10^6$ cells per culture dish) were cultured for 12 h with 2 ml medium of 4% FBS-supplemented RPMI-1640 containing EDTA at the final concentrations of 0.0625, 0.125, 0.25, 0.5, 1 or 2 mmol $l^{-1}$. The soluble E-cadherin concentration of each culture supernatant was measured by the above IEMA method.

### Results

*Characterization of MAbs*

HECD-1 (Shimoyama *et al.*, 1989) and SHE13-6 MAbs were determined to be IgG1 subclasses essentially according to the ELISA method as described previously (Harlow & Lane, 1988). One characteristic feature of the cadherin family protein is a sensitivity to degradation by trypsin, which can be blocked by adding $Ca^{2+}$ to the reaction mixture (Yoshida-Noro *et al.*, 1984). HECD-1 was shown to react with SDS lysates of A431 cells and those pretreated with trypsin plus $Ca^{2+}$, and not to bind to A431 lysates prepared in the absence of $Ca^{2+}$ (Figure 1), demonstrating that HECD-1 MAb was specific for human E-cadherin. In another Western blotting analysis, we also found that SHE13-6 could recognise soluble 80 kDa fragments of E-cadherin isolated from human placenta as well as HECD-1.

**Figure 1** Immunoblotting analysis for E-cadherin isolated from human placenta or cultured cells. Insoluble materials (lanes A–C) and supernatants (lane D) of A431 cells, trypsinised in physiological saline containing 1 mmol $l^{-1}$ EDTA (lane A), trypsinised in 5 mmol $l^{-1}$ calcium chloride (lanes B and D) and extracted in saline containing 5 mmol $l^{-1}$ calcium chloride (lane C) were used as samples for 10% SDS-PAGE. Immunopurified placental E-catherin (lane E) was separated on the same gels, transferred onto the membrane and visualised by the immunoblotting procedure as described in Materials and methods.

**Figure 2** Dilution curves of five serum samples from a healthy subject (●) and patients with gastric tumour (○), hepatocellular tumour (△), colon tumour (■) and leiomyosarcoma (▲).

*IEMA for E-cadherin*

Soluble E-cadherin isolated from human placenta was purified into a homogeneous molecule which migrated as an 80 kDa peptide on SDS-PAGE gel, and was highly reactive to both HECD-1 and SHE13-6 MAbs in the ELISA. Therefore, we fixed this material for use as an assay standard for quantitation of antigen levels in several biological fluids. Soluble E-cadherin antigens in serum samples from 56 healthy individuals were actually detected by the sandwich IEMA using immobilised HECD-1 and enzyme-labelled SHE13-6 (mean ± s.d. 1.99 ± 0.50 $\mu g\ ml^{-1}$). The precision of this IEMA was tested by assaying serum samples obtained from four healthy donors 20 times (intra-assay) and in ten consecutive assays (interassay), and their coefficient of variation values were all under 10%. The dilution curves for the five serum samples appeared to be linear, suggesting that the same immunoreactive substances were accurately measured in the different dilution series (Figure 2). We found that the most moderate measurement for soluble E-cadherin in serum was performed by 40-fold dilution of all the samples from 56 healthy individuals. In the 50 healthy individuals entered in this study, serum E-cadherin levels in 15 subjects with ages ranging from 21 to 30 years (1.85 ± 0.55 $\mu g\ ml^{-1}$) were not significantly lower than those in eight with ages ranging from 31 to 40 (2.14 ± 0.51), those in eight with ages ranging from 41 to 50 (2.04 ± 0.32), those in ten with ages ranging from 51 to 60 (2.23 ± 0.63), those in three with ages ranging from 61 to 70 (2.11 ± 0.24), those in three with ages ranging from 71 to 80 (2.09 ± 0.13) or those in three with ages over 81 (2.15 ± 0.20). We also found that mean level for serum E-cadherin in 21 healthy men was not significantly higher (1.98 ± 0.61) than that in 21 healthy women (1.97 ± 0.46). Serum levels for E-cadherin in the healthy subjects, the non-malignant patients with diabetes mellitus or acute hepatitis and the patients with tumours are shown in Figure 3. As compared with the normal levels obtained above, significantly elevated levels of soluble E-cadherins in serum were observed in the 54 cancer patients (3.80 ± 2.36, $P<0.0001$), but were not in the 26 patients with diabetes mellitus (2.33 ± 1.58). The serum E-cadherin levels in 22 patients with gastric cancers (3.51 ± 1.79 $\mu g\ ml^{-1}$, $P<0.0001$), those in 11 patients with hepatocellular carcinomas (5.55 ± 3.11, $P<0.0001$) and those in ten patients with other cancers (3.93 ± 2.75, $P<0.0001$) were found to be significantly elevated compared with those in 56 healthy subjects. The levels in 11 patients with leukaemia (2.52 ± 1.00, $P<0.01$) were found to be elevated significantly compared with those in 56 healthy subjects, but their values were not significantly higher than those in 26 diabetic patients (Figure 3). In contrast to leukaemic patients, 22 patients with gastric tumour, 11 patients with hepatocellular tumour and ten patients with the other tumours obviously had higher levels of circulating E-cadherin than the diabetic patients ($P<0.02$, $P<0.001$ and $P<0.05$ respectively). The levels in four patients with leiomyosarcoma included in the group of the other cancers were found to be slightly elevated (3.28 ± 0.72 $\mu g\ ml^{-1}$, $P<0.05$) than the normal levels but not significantly higher than those in the diabetic patients. Another patient group with non-malignant hepatitis exhibited normal serum E-cadherin levels (2.34 ± 0.52 $\mu g\ ml^{-1}$), which were almost identical to the levels observed in the diabetic patients.

*Immunoblot analysis for serum E-cadherin*

In immunoblot analysis after 5–20% SDS-PAGE, we initially separated the immunoisolated proteins, reactive with immobilised SHE13-6, from serum samples of three healthy volunteers and six cancer patients who were randomly

**Figure 3** Levels of soluble E-cadherin in sera of 56 healthy subjects, 26 diabetes patients, four hepatitis patients and 54 cancer patients. Serum E-cadherin levels were determined by IEMA. The mean level in each group is indicated by a horizontal bar. NS, not significant.

**Figure 4** Immunoblotting analysis for serum E-cadherin. Lane A, A431 cell lysate; lanes B–G, antigen isolated from sera of six cancer patients; lanes H–J, antigen isolated from sera of three healthy subjects. Each lane (B–J) indicates the immunoreactive antigen from different individuals. Serum E-cadherin was immunoisolated using immobilised SHE13-6 MAb and separated on 5–20% SDS-PAGE. Molecules immunoreactive with peroxidase-labelled HECD-1 were visualised by the colour intensity of substrates according to the standard Western blotting technique.

selected from the subjects used in this study. About 75–80 kDa E-cadherin molecules present in serum were detected by peroxidase-labelled HECD-1 MAbs and substrates (Figure 4). The molecular weights of soluble E-cadherins of several serum samples, from either healthy subjects or cancer patients, were almost all identical (Figure 4, lanes B–J). HECD-1 MAb could also bind to E-cadherins at the approximate molecular weight of 110 kDa in the whole-cell lysate of A431, which consisted of intact membrane-associated molecules (Figure 4, lane A).

*Soluble E-cadherin released from cultured cells*

Physiological concentrations of $Ca^{2+}$ and $Mg^{2+}$ both approximate to 0.4 mmol $l^{-1}$. The release of soluble E-cadherins increased with the elevation of EDTA concentrations, ranging from 0.0625 to 0.5 mmol $l^{-1}$, in the culture medium. Immunoreactive soluble E-cadherins completely disappeared in the culture fluids at EDTA concentrations of 1 mmol $l^{-1}$ or higher, because at these concentrations EDTA was recognised to chelate all of the divalent cations in the culture medium, and E-cadherin on the cell surface was greatly degraded into undetectable smaller fragments (Yoshida & Takeichi, 1982).

### Discussion

E-cadherin was supposed to exist in serum in the monomeric soluble form, because the same concentrations were recovered accurately along the linear dilution curves on the IEMA quantitation (Figure 2). IEMA was applicable basically to the measurement of soluble E-cadherin in citrated plasma, but serum was better for routine use in the cadherin assay, as dissolved $Ca^{2+}$ was not removed artificially by chelation. In the experiments using cultured human cells, we found that a significant amount of soluble E-cadherin was released from the monolayers of living A431 cells by the chelating action of EDTA below physiological concentrations of divalent cations; furthermore, released soluble E-cadherin would fail to react with MAbs under the further enhancement of chelation (Figure 5). We identified from this study, as well as from results obtained previously (Yoshida & Takeichi, 1982), that coexistent $Ca^{2+}$ is necessary to stabilise the structure of soluble E-cadherin immunoreactive with those MAbs.

Several reports have stated that metastatic tumour cells might be less homotypically adhesive than their non-metastatic counterparts, especially in immunological analysis for the homotypic cell–cell adhesion molecules, but their conclusions appear to be conflicting (Lotan & Raz, 1983; Hixson *et al.*, 1985; Reeves, 1992). In contrast to the other cell–cell adhesive receptors, dysfunction in the regulation of cadherin expression might very well be involved in cancer development and progression (Takeichi, 1991; Umbas *et al.*, 1992). Furthermore, E-cadherin is well known to be an invasive suppressor in tumour progressions, because expression levels for E-cadherins were reduced in almost all the malignant tumours and not correlated with their metastatic abilities (Behrens *et al.*, 1989; Hashimoto *et al.*, 1989). Additionally, a recent report suggested the possibility that some carcinoma cells lose E-cadherin expression during the process of detaching from the primary sites and infiltrating other sites (Matsuura *et al.*, 1992); another elucidated that some E-cadherin functions expressed on the cancerous cell surfaces are impaired by loss or down-regulation of cytoplasmic proteins designated 'catenins' (Shimoyama *et al.*, 1992). Here, we understand that E-cadherin dysfunction in tumour cells was partly mediated by the degradation of proteases secreted from these cells, because soluble E-cadherin with a molecular weight of about 80 kDa remarkably increased in the circulation of cancer patients (Figure 4), and it can reasonably be derived from proteolytic digests of the cell-surface E-cadherin.

Healthy individuals appear to have continuous E-cadherin regeneration and to process a small amount of soluble E-cadherin into the blood flow, resulting in a serum E-cadherin concentration of 2 μg $ml^{-1}$ (Figure 3). However, elevated levels of soluble E-cadherin were detected more frequently in

**Figure 5** Release of soluble E-cadherin from cultured A431 cells under several concentrations of EDTA. Monolayers of A431 cells were cultured for 12 h in RPMI-1640 medium containing 4% FBS and EDTA at several final concentrations.

malignant patients but not in the patients with diabetes mellitus or acute hepatitis (Figure 3). Thus, we suggest that proteolytic degradation of E-cadherins in cancers may be associated with malignancy, invasiveness or the metastatic ability of tumour cells at the primary sites. No significant elevation of circulating E-cadherin levels was observed in the studied non-epithelial malignancies, such as leukaemias or leiomyosarcomas, compared with the levels in the benign disease patients. This result can potentially suggest that serum E-cadherin fragments may be a clinical marker specific to detecting epithelial carcinomas. However, the relationship between soluble E-cadherin levels in the circulation and the metastatic potency of tumours needs to be evaluated in further clinical studies, e.g. using experimental organ metastasis in animal models.

Recent immunohistochemical findings of decreased E-cadherin in progressive tumours suggest that some change in the expression level of cell-surface E-cadherin apparently occurs in malignant tissues (Shimoyama & Hirohashi, 1991*b*; Rasbridge *et al.*, 1993). This is the first investigation in which soluble cadherin has been found to be present in human biological fluids. In the present study, we propounded the novel process, which is most frequent in epithelial cancers, of shedding E-cadherin from cell surfaces and releasing its fragments into the circulation. Recent reports have demonstrated that soluble forms of some cell-surface receptors that are increased in the biological fluids of cancer patients might be processed by proteolytic cleavage and retain the functional activities of their original cell-surface molecules (Symons *et al.*, 1988; Rothlein *et al.*, 1991). In our preliminary observation, serum E-cadherin also interfered with cell–cell adhesion in the cultured A431 cells at a final concentration of $50\,\mu g\,ml^{-1}$, and the disruptive effect of purified soluble E-cadherin is reversible because the cells resumed their normal morphology after removal of supplemented soluble E-cadherin (M. Katayama *et al.*, manuscript in preparation). Therefore, soluble E-cadherin fragments released from the cell surface constitute a mode of down-regulation of the cell-to-cell adhesiveness mediated by E-cadherin, resulting in decreased responsiveness to the adjacent cell-surface E-cadherin. The biological potency of soluble E-cadherin released from cultured cells has already been characterised in *in vitro* experiments (Wheelock *et al.*, 1987).

Some $\beta_1$-integrins have been shown to function as intercellular adhesion molecules in *in vitro* cultured keratinocytes (Larjava *et al.*, 1990), and therefore the function or *in vivo* localisation of integrin $\beta_1$ in tumour cells is often compared with that of cadherins using immunological analysis (Pignatelli *et al.*, 1992). Many reports have suggested, contrary to the findings for E-cadherin, that the surface expression of integrin $\beta_1$ is uniformly elevated in metastatic tumour cells and potentially mediates the tight adhesion of these cells to the subendothelial matrix substratum (Feldman *et al.*, 1991; Martin-Padura *et al.*, 1991). It has already been reported that soluble integrin $\beta_1$ is present in the human circulation and that serum levels of soluble integrin $\beta_1$ are unlikely to be elevated in cancer patients (Katayama *et al.*, 1991). We observed non-correlation between soluble integrin $\beta_1$ and soluble E-cadherin fragments in the serum of the studied cancer patients (data not shown). On the other hand, soluble laminin in biological fluids was demonstrated to be mostly fragmented and sometimes used as a tumour marker (Brocks *et al.*, 1986; Katayama *et al.*, 1992). Serum laminin levels were significantly correlated with serum E-cadherin in the cancer patients (data not shown). This fact could suggest that degradation, release and shedding of E-cadherin on the tumour cells were somehow related to the proteolytic action by those cells, required for penetrating the extracellular matrix containing laminin or collagen (Goldfarb & Liotta, 1986).

Recently, catenin has been recognised as a most important regulator of cadherin function (Nagafuchi *et al.*, 1991). Although the cadherin–catenin association is lost in some tumour cell lines or disturbed by oncogenic transformation (Shimoyama *et al.*, 1992; Hamaguchi *et al.*, 1993), there are no data available with regard to the prevailing mechanisms responsible for decreased E-cadherin staining in human tumour tissues (Shimoyama & Hirohashi, 1991*b*; Rasbridge *et al.*, 1993). One of the answers to this could be supplied by the previous observation that a metastatic phenotype is induced by the genetic deletion of E-cadherin as an invasion suppressor (Chen & Öbrink, 1991; Vleminckx *et al.*, 1991). It is possible that E-cadherin degradation and shedding from the tumour cell surface is due to the increase in fragmented E-cadherin in the soluble form in the circulation of cancer patients, independent of the genetic abnormalities of cadherin or cadherin-associated molecules.

In conclusion, it is evident that soluble E-cadherin fragment in serum, detected by the two-site IEMA investigated by us for the first time, can be used as an innovative tumour marker. The levels for serum E-cadherin fragments may be used to accurately reflect the progressive regeneration of E-cadherin around epithelial tumours, and probably to monitor the recurrence of cancer patients after surgical removal of primary tumours, or to evaluate the effects of anti-cancer treatments.

We thank Dr Makoto Handa (Blood Center, Keio University Hospital, Tokyo, Japan) for providing serum samples in this clinical study.

### References

BEHRENS, J., MAREEL, M.M., VAN ROY, F.M. & BIRCHMEIER, W. (1989). Dissecting tumor cell invasion: epithelial cells acquire invasive properties after the loss of uvomorulin-mediated cell-cell adhesion. *J. Cell Biol.*, **108**, 2435–2447.

BROCKS, D.G., STRECKER, H., NEUBAUER, H.P. & TIMPLE, R. (1986). Radioimmunoassay of laminin in serum and its application to cancer patients. *Clin. Chem.*, **32**, 787–791.

BUSSEMAKERS, M.J.G., VAN MOORSELAAR, R.J.A., GIROLDI, L.A., ICHIKAWA, T., ISAACS, J.T., TAKEICHI, M., DEBRUYNE, F.M.J. & SCHALKEN, J.A. (1992). Decreased expression of E-cadherin in the progression of rat prostatic cancer. *Cancer Res.*, **52**, 2916–2922.

CHEN, W. & ÖBRINK, B. (1991). Cell-cell contacts mediated by E-cadherin (uvomorulin) restrict invasive behavior of L-cells. *J. Cell Biol.*, **114**, 319-327.

DAMSKY, C.H., RICHA, J., SOLTER, D., KNUDSEN, K. & BUCK, C.A. (1983). Identification and purification of a cell surface glycoprotein mediating intercellular adhesion in embryonic and adult tissue. *Cell*, **34**, 455-466.

FELDMAN, L.E., SHIN, K.C., NATALE, R.B. & TODD, III, R.F. (1991). β1 integrin expression on human small cell lung cancer cells. *Cancer Res.*, **51**, 1065-1070.

FRIXEN, U.H., BEHRENS, J., SACHS, M., EBERLE, G., VOSS, B., WARDA, A., LÖCHNER, D. & BIRCHMEIER, W. (1991). E-cadherin-mediated cell-cell adhesion prevents invasiveness of human carcinoma cells. *J. Cell Biol.*, **113**, 173-185.

GIARD, D.J., AARONSON, S.A., TODARO, G.J., ARNSTEIN, P., KERSY, J.H., DOSIK, H. & PARK, W.P. (1973). *In vitro* cultivation of human tumors: establishment of cell lines derived from a series of solid tumors. *J. Natl Cancer Inst.*, **51**, 1417-1423.

GOLDFARB, R.H. & LIOTTA, L.A. (1986). Proteolytic enzymes in cancer invasion and metastasis. *Semin. Thromb. Hemost.*, **12**, 294-307.

HAMAGUCHI, M., MATSUYOSHI, N., OHNISHI, Y., GOTOH, B., TAKEICHI, M. & NAGAI, Y. (1993). $p60^{v\text{-}src}$ causes tyrosine phosphorylation and inactivation of the N-cadherin-catenin cell adhesion system. *EMBO J.*, **12**, 307-314.

HARLOW, E. & LANE, D. (1988). *Antibodies: A Laboratory Manual.* Cold Spring Harbor Laboratory Press: Cold Spring Harbor, NY.

HASHIMOTO, M., NIWA, O., NITTA, Y., TAKEICHI, M. & YOKORO, K. (1989). Unstable expression of E-cadherin adhesion molecules in metastatic ovarian tumor cell. *Jpn J. Cancer Res.*, **80**, 459-463.

HIXSON, D.C., MCENTIRE, K.D. & ÖBRINK, B. (1985). Alterations in the expression of a hepatocyte cell adhesion molecule by transplantable rat heptocellular carcinomas. *Cancer Res.*, **45**, 3742-3749.

KATAYAMA, M., KUROME, T., YAMAMOTO, K., UCHIDA, H., HINO, F. & KATO, I. (1991). Sandwich enzyme immunoassay for serum integrins using monoclonal antibodies. *Clin. Chim. Acta*, **202**, 179-190.

KATAYAMA, M., KAMIHAGI, K., HIRAI, S., KUROME, T., MURAKAMI, K., HINO, F. & KATO, I. (1992). Urinary laminin fragments as a tumour marker potentially reflecting basement membrane destruction. *Br. J. Cancer*, **65**, 509-514.

LARJAVA, H., PELTONEN, J., AKIYAMA, S.K., YAMADA, S.S., GRALNICK, H.R., UITTO, J. & YAMADA, K.M. (1990). Novel function for β1 integrins in keratinocyte cell-cell interactions. *J. Cell Biol.*, **110**, 803-815.

LOTAN, R. & RAZ, A. (1983). Low colony formation *in vivo* and in culture as exhibited by metastatic melanoma cells selected for reduced homotypic aggregation. *Cancer Res.*, **43**, 2088-2093.

MAREEL, M.M., BEHRENS, J., BIRCHMEIER, W., DE BRUYNE, G.K., VLEMINCKX, K., HOOGEWIJS, A., FIERS, W.C. & VAN ROY, F.M. (1991). Down-regulation of E-cadherin expression in Madin Darby canine kidney (MDCK) cells inside tumors of nude mice. *Int. J. Cancer*, **47**, 922-928.

MARTIN-PADURA, I., MORTARINI, R., LAURI, D., BERNASCONI, S., SANCHEZ-MADRID, F., PARMIANI, G., MANTOVANI, A., ANICHINI, A. & DEJANA, E. (1991). Heterogeneity in human melanoma cell adhesion to cytokine activated endothelial cells correlates with VLA-4 expression. *Cancer Res.*, **51**, 2239-2241.

MATSUURA, K., KAWANISHI, J., FUJII, S., IMAMURA, M., HIRANO, S., TAKEICHI, M. & NIITSU, Y. (1992). Altered expression of E-cadherin in gastric cancer tissues and carcinomatous fluid. *Br. J. Cancer*, **66**, 1122-1130.

NAGAFUCHI, A., TAKEICHI, M. & TSUKITA, S. (1991). The 102 kd cadherin-associated protein: similarity to vinculin and posttranscriptional regulation of expression. *Cell*, **65**, 849-857.

NAVARRO, P., GOMEZ, M., PIZARRO, A., GAMALLO, C., QUINTANILLA, M. & CANO, A. (1991). A role for the E-cadherin cell-cell adhesion molecule during tumor progression of mouse epidermal carcinogenesis. *J. Cell Biol.*, **115**, 517-533.

OGOU, S., YOSHIDA-NORO, C. & TAKEICHI, M. (1983). Calcium-dependent cell-cell adhesion molecules common to hepatocytes and teratocarcinoma cells. *J. Cell Biol.*, **97**, 944-948.

PIGNATELLI, M., LIU, D., NASIM, M.M., STAMP, G.W.H., HIRANO, S. & TAKEICHI, M. (1992). Morphoregulatory activities of E-cadherin and beta-1 integrins in colorectal tumour cells. *Br. J. Cancer*, **66**, 629-634.

RASBRIDGE, S.A., GILLETT, C.E., SAMPSON, S.A., WALSH, F.S. & MILLIS, R.R. (1993). Epithelial (E-) and placental (P-) cadherin cell adhesion molecule expression in breast carcinoma. *J. Pathol.*, **169**, 245-250.

REEVES, M.E. (1992). A metastatic tumor cell line has greatly reduced levels of a specific homotypic cell adhesion molecule activity. *Cancer Res.*, **52**, 1546-1552.

ROTHLEIN, R., MAINOLFI, E.A., CZAJKOWSKI, M. & MARLIN, S.D. (1991). A form of circulating ICAM-1 in human serum. *J. Immunol.*, **147**, 3788-3793.

SCHIPPER, J.H., FRIXEN, U.H., BEHRENS, J., UNGER, A., JAHNKE, K. & BIRCHMEIER, W. (1991). E-cadherin expression in squamous cell carcinomas of head and neck: inverse correlation with tumor dedifferentiation and lymph node metastasis. *Cancer Res.*, **51**, 6328-6337.

SHIMOYAMA, Y. & HIROHASHI, S. (1991*a*). Expression of E- and P-cadherin in gastric carcinomas. *Cancer Res.*, **51**, 2185-2192.

SHIMOYAMA, Y. & HIROHASHI, S. (1991*b*). Cadherin intercellular adhesion molecule in hepatocellular carcinomas: loss of E-cadherin expression in an undifferentiated carcinoma. *Cancer Lett.*, **57**, 131-135.

SHIMOYAMA, Y., HIROHASHI, S., HIRANO, S., NOGUCHI, M., SHIMOSATO, Y., TAKEICHI, M. & ABE, O. (1989). Cadherin cell-adhesion molecules in human epithelial tissues and carcinomas. *Cancer Res.*, **49**, 2128-2133.

SHIMOYAMA, Y., NAGAFUCHI, A., FUJITA, S., GOTOH, M., TAKEICHI, M., TSUKITA, S. & HIROHASHI, S. (1992). Cadherin dysfunction in a human cancer cell line: possible involvement of loss of α-catenin expression in reduced cell-cell adhesiveness. *Cancer Res.*, **52**, 5770-5774.

SUZUKI, S., SANO, K. & TANIHARA, H. (1991). Diversity of the cadherin family: evidence for eight new cadherins in nervous tissue. *Cell Reg.*, **2**, 261-270.

SYMONS, J.A., WOOD, N.C., DI GIOVINE, F.S. & DUFF, G.W. (1988). Soluble IL-2 receptor in rheumatoid arthritis: correlation with disease activity, IL-1 and IL-2 inhibition. *J. Immunol.*, **141**, 2612-2618.

TAKEICHI, M. (1988). The cadherins: cell-cell adhesion molecules controlling animal morphogenesis. *Development*, **102**, 639-655.

TAKEICHI, M. (1991). Cadherin cell adhesion receptors as a morphogenetic regulator. *Science*, **251**, 1451-1455.

UMBAS, R., SCHALKEN, J.A., AALDERS, T.W., CARTER, B.S., KARTHAUS, H.F.M., SCHAAFSMA, H.E., DEBRUYNE, F.M.J. & ISAACS, W.B. (1992). Expression of the cellular adhesion molecule E-cadherin is reduced or absent in high-grade prostate cancer. *Cancer Res.*, **52**, 5104-5109.

VESTWEBER, D. & KEMLER, R. (1984). Rabbit antiserum against a purified surface glycoprotein decompacts mouse preimplantation embryos and reacts with specific adult tissues. *Exp. Cell Res.*, **152**, 169-178.

VLEMINCKX, K., VAKAET, L., MAREEL, M., FIERS, W. & VAN ROY, F. (1991). Genetic manipulation of E-cadherin expression by epithelial tumor cells reveals an invasion suppressor role. *Cell*, **66**, 107-119.

WHEELOCK, M.J., BUCH, C.A., BECHTOL, K.B. & DAMSKY, C.H. (1987). Soluble 80-kd fragment of cell-CAM 120/80 disrupts cell-cell adhesion. *J. Cell Biochem.*, **34**, 187-202.

YOSHIDA, C. & TAKEICHI, M. (1982). Teratocarcinoma cell adhesion: identification of a cell-surface protein involved in calcium-dependent cell aggregation. *Cell*, **28**, 217-224.

YOSHIDA-NORO, C., SUZUKI, N. & TAKEICHI, M. (1984). Molecular nature of the calcium-dependent cell-cell adhesion system in mouse teratocarcinoma and embryonic cells studied with a monoclonal antibody. *Dev. Biol.*, **101**, 19-27.